# Health risk assessment of PM_2.5_ on walking trips

**DOI:** 10.1038/s41598-021-98844-6

**Published:** 2021-09-28

**Authors:** Caihua Zhu, Zekun Fu, Linjian Liu, Xuan Shi, Yan Li

**Affiliations:** College of Transportation Engineering, Chang’an University, Middle section of south 2nd Ring Road, Xi’an, 710064 Shaanxi Province China

**Keywords:** Environmental social sciences, Risk factors

## Abstract

PM_2.5_ has an impact on residents' physical health during travelling, especially walking completely exposed to the environment. In order to obtain the specific impact of PM_2.5_ on walking, 368 healthy volunteers were selected and they were grouped according to gender and age. In the experiment, the heart rate change rate (HR%) is taken as test variable. According to receiver operating characteristic (ROC) curve, the travel is divided into two states: safety and risk. Based on this, a binary logit model considering Body Mass Index (BMI) is established to determine the contribution of PM_2.5_ concentration and body characteristics to travel risk. The experiment was conducted on Chang'an Middle Road in Xi'an City. The analysis results show that the threshold of HR% for safety and risk ranges from 31.1 to 40.1%, and that of PM_2.5_ concentration ranges from 81 to 168 μg/m^3^. The probability of risk rises 5.8% and 11.4%, respectively, for every unit increase in PM_2.5_ concentration and HR%. Under same conditions, the probability of risk for male is 76.8% of that for female. The probability of risk for youth is 67.5% of that for middle-aged people, and the probability of risk for people with BMI in healthy range is 72.1% of that for non-healthy range. The research evaluates risk characteristics of walking in particular polluted weather, which can improve residents’ health level and provide suggestions for travel decision while walking.

## Introduction

The health risks in residents' travel are mainly related to the concentration of particulate matter (PM)^1^. PM is the primary cause of many adverse health effects, including but not limited to cardiovascular and respiratory diseases^2,3^. As the main PM exposure environment for residents, traffic environment involves a large number of people. PM has a more significant impact on commuters with intensive travel time and high travel demand^4^. Walking as the final transfer mode in a trip is an indispensable part to complete the travel. Since walking is fully exposed to the external environment, it is very sensitive to the change of PM concentration. In order to assess the health risk of walking trips, we need to address two aspects: (1) The human body has its own regulatory capacity. How to determine the boundary between the safe state and the risk state? (2) The quantitative description of the factors that influence the health risk of travel is not clear. If the health risk degree at certain PM concentration could be obtained, the health risk degree, i.e., the probability of health risks when residents travel in specific polluted weather. For commuters, it will be possible to alter residents' willingness to travel or their travel modes, and improve the health conditions in travelling. For managers, it will provide risk assessment basis for health control, and make commuters' travel reach the overall optimal level.

The health risks during commuting are not only closely relevant to the concentration of pollutants in the external environment, but also connected to residents' personal physical conditions. Due to people’s different physical qualities, their resistance to pollution is not always the same^5^. Individual characteristics (age, gender, figure and so on) have an effect on physical quality^6,7^. When estimating the critical value of pollutant concentration corresponding to safety and risk, individual situations of each volunteer should be analyzed. The health risk of walking trips in polluted weather can be reflected by a variety of factors. Among them, real-time physiological data during travel is the most active reflection to the disturbance of external environment. Heart rate as the most intuitive sample data has high reliability since it is less influenced by commuters' subjective consciousness^8^. Thus, the impact of pollutant concentration on residents' health can be measured by the changes of heart rate. The health status is affected by a combination of the external environment and physical condition. So, it is necessary to study the specific contribution of each influencing factor to health risk. Because the experiments were conducted simultaneously, the analysis was carried out from the perspective of PM concentration. The novelty of this study lies in obtaining health risk thresholds for volunteers in terms of heart rate and quantitatively describing the relationship between each influencing factor and health risk degree.

The sources of PM include natural and anthropogenic sources. Natural sources mainly include dust storms, volcanic eruptions, forest and grassland fires; anthropogenic sources mainly include traffic trips, home heating, power plants and various industrial processes^5^. Due to the numerous natural and anthropogenic sources, PM may have different physicochemical characteristics in different areas and it is diffuse in nature^5,9^. PM concentrations show seasonality, with higher average PM concentrations in the warm season than in the cool season, and higher PM concentrations from June to September^7,10^. Meanwhile, factors such as distance from dust source, frequency and duration of dust storms, vegetation cover, and soil type also have a greater influence on PM concentrations^7^. In areas of human activity, PM concentrations are accompanied by spatial shifts that are related to the frequency of human activity^11^. The increased concentration of PM to which individuals are exposed may alter metabolic activity and lead to serious diseases such as asthma, rhinitis, and tuberculosis^12^. The number of hospitalizations for respiratory problems shows a positive correlation with PM concentrations^13^.

PM concentrations are generally collected with the help of sampling instruments to monitor individual exposure levels^14^. There are three main methods in the research of PM concentration. The first method is to estimate the impact by measuring PM concentration in the environment. It is a kind of intuitive statistics with single environmental variable data, which is often used to compare PM concentrations in different regions^15^. The second method is to establish a dose model through breathing frequency to estimate the suction volume of pollutant. Then the size of suction volume is adopted to determine the degree of risk. This type of method is often used in the comparison of different travel modes^16,17^. The last method is to evaluate the impact on human health by combining PM concentrations with physiological indicators. Most of collected statistics are time series data, which can illustrate the variation tendency of physiological data with PM concentration changes^18,19^. Commonly used physiological indicators are electrocardiogram (ECG) and electroencephalogram (EEG)^20^. ECG is more widely used due to its simple and accurate measurement^21^. Heart rate and heartbeat interval^22^ are highly stable and they could visualize changes in the external environment. So, these two indexes are usually applied to describe health effects caused by PM concentrations.

In the statistics of travel health risk, PM concentrations are main considerations in the degree of risk. The higher the PM concentrations, the greater the risk. Common approaches in identifying risks include intuitive statistics^23^, Markov model^24^, Strengths Weaknesses Opportunities Threats (SWOT)^25^, Receiver Operating Characteristic (ROC) curves^26^ and so on. Judging health risk actually lies in the determination of threshold for safety and risk in the travel. ROC curves as a series of different binary classification methods are able to calculate critical value of risk. High classification accuracy of ROC curves leads to their widespread application in the field of medical image recognition and disease recognition^27^. Intuitive analysis models for PM focus on linear probability models and discrete probability models. The former is mainly used to connect PM to health indicators^28^. The latter is adopted to calculate the probability of a specific event, among which Logit model and Probit model are representative models. Logit model has a faster solution speed as a derivative of discrete choice models^4^. This research is based on the analysis of binary classification for safety and risk, so binary logit model can be selected to model and analyze influencing factors of health during travelling. Previous studies (Table 1) have shown that PM has an impact on human health, and this impact is related to exposure to the environment and physical conditions.

**Table 1:** Summary of relevant past the characteristics of PM concentration distribution and the relationship between PM concentration and health risk.

Study area (place)	Pollutant types	Key observations	Author (year)
Chile	PM_2.5_	Personal PM exposure concentration and its influencing factors of commuters with different transportation modes	Suárez, L. et al. (2014)
Iran	PM_2.5_/PM_10_	Concentrations of annual PM exceeding the WHO air quality guideline, and an unacceptably high risk for human health	Yunesian, M. et al. (2019)
China	PM_2.5_	Short-term exposure to ambient PM2.5 was significantly associated with an increased risk of daily outpatient visits for ulcerative colitis, and related to gender and age	Duan, R. et al. (2021)
Iran	PM_10_	The average PM10 concentration was higher in summer. Higher exposure levels in female	Ahmadi, S. et al. (2021)
Iran	PM	Most of particles were inorganic in nature, and PM may have different physicochemical characteristics in different areas	Sajjadi, S. A. et al. (2018)
Iran	PM_2.5_/PM_10_	The PM concentration was higher in the warm season than in the cool season, and the number of colonies increased with the increase in PM concentration	Amarloei, A. et al. (2020)
India	PM_1_/PM_2.5_/PM_10_	PM concentrations are accompanied by spatial shifts that are related to the frequency of human activity	Sahu, V. et al. (2018)
India	PM_2.5_/PM_10_	The number of hospitalizations for respiratory problems shows a positive correlation with PM concentrations, and PM10 has 2 times more impact on human health than PM2.5	Gupta, A. et al. (2019)
China	PM_2.5_	The spatial distribution of PM2.5 concentration in Xi’an and the building distribution does not match	Sun, X. et al. (2020)

PM concentrations have an impact on the health of residents who travel walking. However, PM concentration thresholds that create risk and the quantitative description of associated influencing factors remain unknown. The objective of this study is to obtain the probability of health risk caused by walking in polluted weather based on actual experimental investigations. Major contributions of the research include: (1) the health risk threshold of each volunteer is determined with heart rate indicators as input variables; (2) binary logit model of health risk is established with safety and risk as defined interval, and the relationship between influencing factors and health risk is quantitated, the factors that contributed most to health risk are determined. The paper adds to the current literature by quantitatively describing the effects of PM on human health. This study adds a health risk threshold jointly determined by PM concentration and heart rate indicator, and a quantitative description of the relationship between influencing factors and health risk with the help of a dichotomous approach, providing basic theoretical knowledge for the detection of health risks in other travel modes.

## Methods

### Framework

Commuters’ physical functions vary from person to person, and the same environmental factors have different effects on human health. So, it is necessary to make a concrete analysis considering commuters’ own situations. PM concentration will not impact health extensively in a certain range due to the self-adjustment function of human body. Only if it is higher than a fixed value can this impact be reflected. Therefore, it is required to find out this critical value in the analysis of health risk.

Identifying safe and risk state belongs to binary classification problem. ROC curve as a binary classification machine learning algorithm can intuitively reflect measurement accuracy of the model at different thresholds and effectively solve binary classification problem. Note that the process of determining thresholds between safe and risk state is conducted for each volunteer independently. While studying the effect of PM concentration on travel health, relying solely on qualitative descriptions has no actual meaning. This raises the need for a relationship model between PM concentration and health risk. According to health risk thresholds, safe state and risk state could be defined during travelling. Then binary logit model can be applied in establishing the relationship model to quantitatively obtain residents' travel health risk under different PM concentrations.

### ROC curve

ROC curve is applicable in binary classification cases, that is, to judge two kinds of results "yes" or "no". ROC curve is a kind of curve that describes the relationship between sensitivity (or True Positive Rate, TPR) and specificity (or False Positive Rate, FPR) with a conformational method in the test^29^. AUC (Area Under ROC Curve) reflects the accuracy of threshold and its value ranges from 0.5 to 1.0. When AUC is greater than 0.9, the accuracy is high. Each point on ROC curve is obtained by changing the classification thresholds in the same model. Thus, the process of determining the best point on the same ROC curve is exactly the process of determining the best classification threshold. If the point is near to the upper left, it has better discriminative effect^30^. In the application, the point with the largest Youden index (Youden index = sensitivity + specificity—1) can be selected as the discriminative threshold on the curve.

In this study, the experimental process is organized as follows:

1. According to the subjective judgment by volunteers after the experiment, PM concentrations are divided into initial safe concentration and risk concentration as the state variable of the model.
2. With HR% as the test variable, basic data points are selected. Then the threshold interval in each calculation is estimated according to the input HR%, and the initial value vector is constructed.
3. Based on discrimination results, Z_TP_ (risk samples and judged as risk samples; number of correctly affirmed matches), Z_FN_ (risk samples but judged as safe samples; number of misses, not correctly found matches), Z_FP_ (safe samples but judged as risk samples; false positives, number of incorrect matches), Z_TN_ (safe samples and judged as safe samples; number of correctly rejected non-matches) corresponding to each threshold are calculated.
4. The values of sensitivity (TPR) and specificity (FPR) are calculated, where $T = {{Z_{TP} } \mathord{\left/ {\vphantom {{Z_{TP} } {(Z_{TP} + Z_{FN} )}}} \right. \kern-\nulldelimiterspace} {(Z_{TP} + Z_{FN} )}}$,$F = {{Z_{FP} } \mathord{\left/ {\vphantom {{Z_{FP} } {(Z_{FP} + Z_{TN} )}}} \right. \kern-\nulldelimiterspace} {(Z_{FP} + Z_{TN} )}}$.
5. Connect the points (*F, T*) of each threshold. Then plot ROC curve and calculate Youden index. The feature point with the largest Youden index on the upper left can be distinguished. HR% corresponding to this feature point is the discriminative threshold of health risk.

### Model construction considering BMI

As a probabilistic model, logit model can predict the probability of an event^31^. This research is related to a binary-state variable reflecting safe and risk state during travelling, so it can be analyzed by binary logit model. In binary logit model, the dependent variable can only take two values 1 and 0 (virtual dependent variable). In this study, the model can not only estimate whether a volunteer is in a risk state, but also calculate the degree of risk at that time. The model are formulated as:

1 $$\hat{Y} = \ln \frac{p}{1 - p} = \beta_{0} + \sum\limits_{i = 1}^{n} {\beta_{i} X_{i} } ,i \in N$$

where, $\hat{Y}$ is the virtual dependent variable and its value is 0 or 1; $p$ is the probability of risk and its value ranges from 0 to 1; $\beta_{0}$ is the undetermined coefficient; $\beta_{i}$ is the coefficient corresponding to the *i*-th explanatory variable; $X_{i}$ is the *i*-th explanatory variable.

The operation of model consists of two parts. The first part is to solve the coefficient of explanatory variable according to virtual dependent variable $\hat{Y}$. The second part is to deduce the probability of risk $p$ based on the variable coefficient and the value of explanatory variable. The probability of risk is calculated as:

2 $$p = {{\exp \left( {\beta_{0} + \sum\limits_{i = 1}^{n} {\beta_{i} X_{i} } } \right)} \mathord{\left/ {\vphantom {{\exp \left( {\beta_{0} + \sum\limits_{i = 1}^{n} {\beta_{i} X_{i} } } \right)} {\left[ {1 + \exp \left( {\beta_{0} + \sum\limits_{i = 1}^{n} {\beta_{i} X_{i} } } \right)} \right]}}} \right. \kern-\nulldelimiterspace} {\left[ {1 + \exp \left( {\beta_{0} + \sum\limits_{i = 1}^{n} {\beta_{i} X_{i} } } \right)} \right]}}$$

Commuters will eventually turn to different risk states due to their different physical conditions even in the same environment. Body Mass Index (BMI) is a commonly used standard to measure the degree of obesity and health. For comparing and analyzing the health effects of weight on people with different heights, BMI is a neutral and reliable index.

In the process of experiment, BMI index is introduced as one of explanatory variables to measure volunteers' health information. This approach aims to avoid the impact of health disparities on model accuracy. The calculation formula of BMI is shown as:

3 $$BMI = \frac{BW}{{H^{2} }}$$

where: *BW* is the weight with unit of kg; *H* is the height with unit of m. For healthy people, their BMI should be in the range of 18.5 to 24.

## Experiments

### Experimental design and path selection

In all procedures, we adhered to the declaration of Helsinki, and the study was received ethics approval from the Ethics Committee of Chang’an University. All participants are over 18 years old. We obtained informed consent from all study participants who were enrolled in the study, and participants were free to leave the experiment at any stage if they felt uncomfortable.

All experiments in this research were conducted during peak hours (7:30 to 8:30 and 17:30 to 18:30) from September to December (fall and winter) in 2019. The research objects were travel processes of volunteers. Since weather had a great influence on the assessment of air pollution concentrations, no tests were arranged in rainy and snowy days. In the selection of volunteers, they were required to be physically and mentally healthy without history of cardiovascular disease, pulmonary disease, nervous system disease and some others. Taking medicines was also not allowed. Then all candidates had a full medical examination to prevent data error caused by physical diseases. Finally, 368 healthy volunteers were selected for the test. All volunteers were grouped according to age and gender. On the basis of classification criteria proposed by World Health Organization^32^, volunteers were divided into 112 young men, 97 young women, 85 middle-aged men and 74 middle-aged women. All participants should rest well in the night before the experiment. Tobacco, wine, tea, coffee, any other food or drugs that might affect volunteer physical status were forbidden in the previous day. Walking is assigned as their travel mode. Before the experiment, the heart rate under relaxed state was measured for 10 min in an air-purified space, which is considered as a healthy heart rate in a pollution-free environment. At the end of experiment, volunteers were asked to complete a subjective questionnaire to determine whether they stayed in risk state at a certain PM concentration.

**Figure 1:**
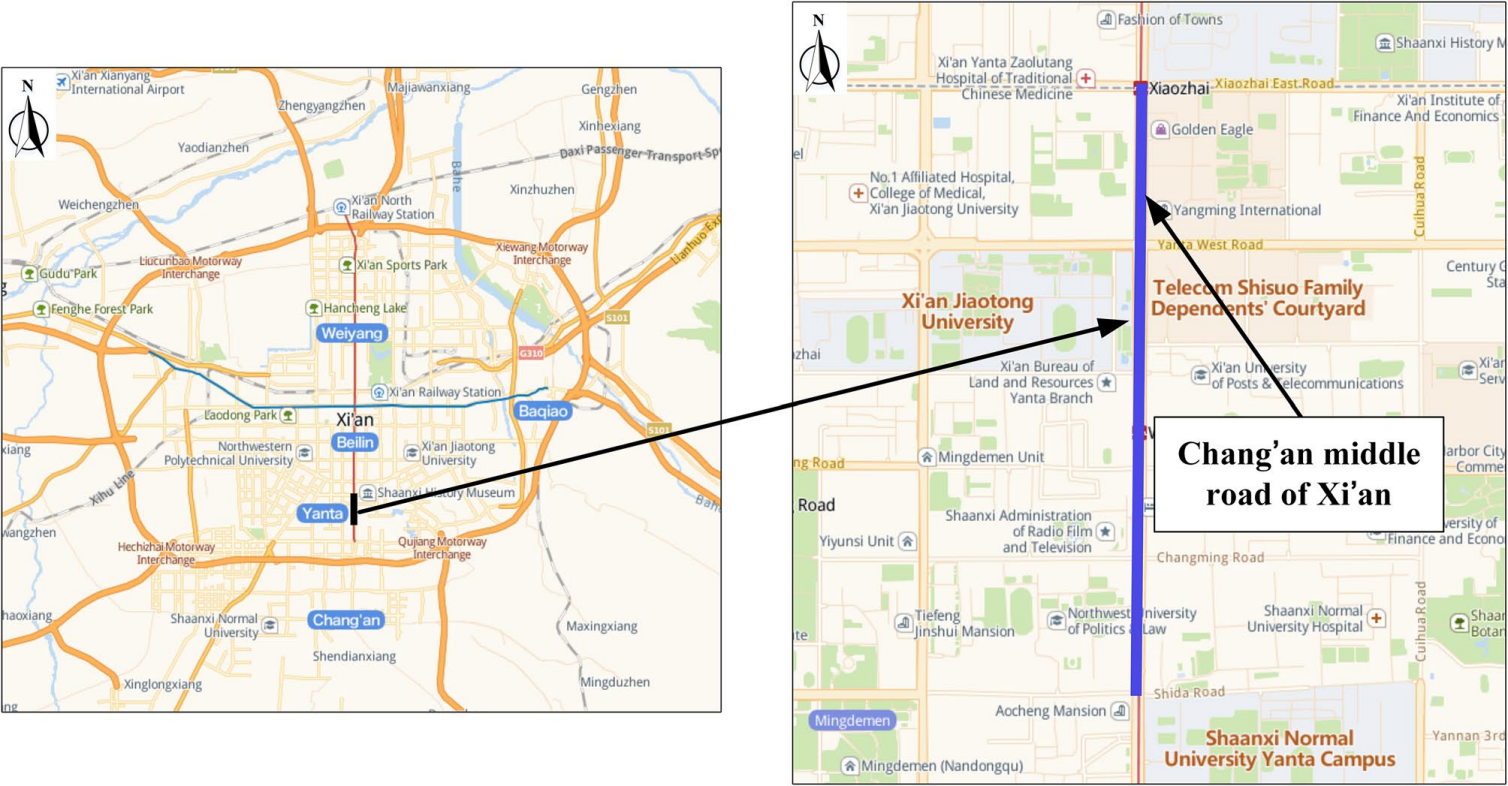
Location of experimental path and its details. And figure was plotted by Microsoft Office Visio 2013, which can be downloaded on https://www.microsoft.com.

Middle Chang'an Road in Xi'an, China, was chosen as a typical experimental path where motor and non-motor vehicles are separated and exclusive sidewalks are set (Fig. 1). The experiment was conducted on the sidewalk. Located in the southern part of Xi'an, this arterial road passes through Xiaozhai business district, high-density residential areas and educational areas. The total length of experimental path is 2 km. The widths of sidewalks range from 3 to 6 m. During the test, average motor vehicle volume on this section was 2662 vehicles/h, consisting of 79% private cars, 12% taxis, 7% buses and less than 2% trucks. The volume of non-motor vehicles was 3008 vehicles/h, containing 42% electric vehicles and 58% bicycles. Pedestrian flow was 1127 people/h.

### Equipment

CEM-DT96 portable particle detector, which supports dual mode of collecting particle concentration and mass concentration, is adopted to detect PM concentration. The sampling interval is set to 1 min considering that walking speed is relatively slow. Each volunteer wears a GARMIN heart rate band with an external GPS module. The equipment continuously records heart rate data and time data in seconds. GARMIN heart rate band is composed of thin film electrode and fabric band, which is worn under the chest line and stick to the skin. Compared with electronic bracelet, GARMIN heart rate band has higher accuracy. In order to guarantee the accuracy and effectiveness of data, the equipment needs to be calibrated synchronously in the same environment before each experiment. All time points take GARMIN detector as reference to ensure the consistency of data and facilitate subsequent data processing.

In the experiment, all volunteers wore GARMIN heart rate band tuned to uniformity and held a qualified CEM-DT96 portable particle detector in their hands to measure heart rate and PM concentration during the walking condition. Heart rate data can be directly exported from the heart rate band, and PM concentration is obtained by directly recording data on the portable particle detector.

### Data processing

**Figure 2:**
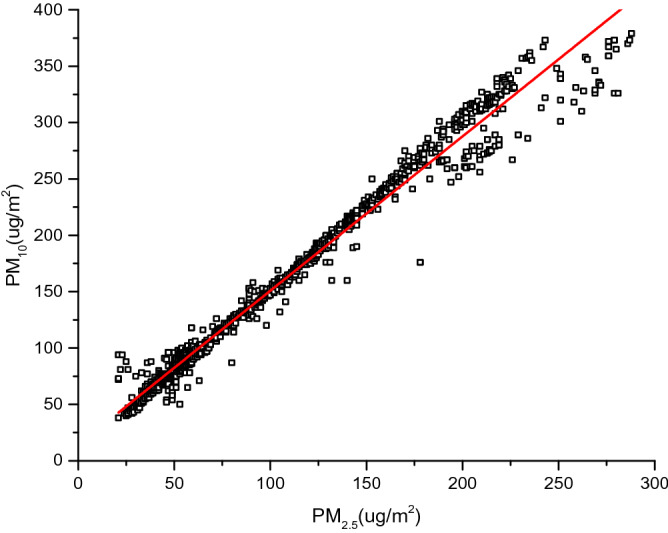
Scatter diagram of relationship between PM_2.5_ and PM_10_.

Collected PM concentrations, including PM_2.5_ concentration and PM_10_ concentration, were derived from the particulate matter detector. Note that sampling time is supposed to correspond to that of GARMIN heart rate band. PM_2.5_ and PM_10_ data represents pollutant concentrations in the external environment. PM_2.5_ and PM_10_ retain a highly positive correlation, as illustrated in Fig. 2. The particle diameter of PM_2.5_ is smaller, so it is easy to be affected by air buoyancy and difficult to sink^33^. As a result, its impact on the atmospheric transparency lasts longer. At the same time, PM_2.5_ can enter the lungs and lead to the reaction of body function, which is more harmful to human than PM_10_^34^. Therefore, PM_2.5_ is analyzed in the modeling of health risk.

The changes of heart rate reflect subtle changes of people in different conditions, which is the basic performance of external environment acting on human. Consequently, it is essential to analyze the relationship between the changes of heart rate and PM_2.5_ concentration. In data statistics, indicators related to heart rate are calculated in seconds.

## Results

### Data statistics

In statistical analysis of data, data set interval has an impact on the accuracy of results and determines the workloads of data processing. If the time interval is too small, the heart rate data will have greater randomness, which tends to highlight some random fluctuations. On the contrary, large intervals may bring about inadequate display of the overall trend and lower accuracy of heart rate changes. As shown in Table 2, significance F (sig. F) and F value reflected in the changes of heart rate data among different samples can be used to find out the appropriate time interval. In statistics, sig. F is the probability that the null hypothesis cannot be rejected in proposed model. F value is a ratio computed via dividing average regression sum of squares by average error sum of squares. The smaller the sig. F (should be less than 0.05) and F value are, the better performance will be. Through integrated into account, the time interval is determined to be 60 s in data processing.

**Table 2:** Sig. F and F values at different time intervals.

Time interval	30 s	60 s	120 s	180 s	240 s	300 s
Sig. F	0.009	0.011	0.027	0.062	0.115	0.136
F	126.3	62.1	58.3	52.0	47.5	38.2

**Figure 3:**
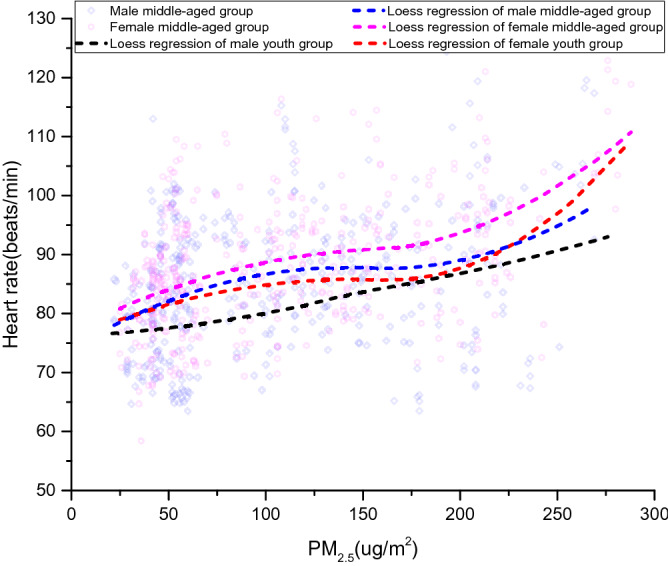
Variation tendency of heart rate.

For each group of volunteers, the relationship between heart rate and PM_2.5_ concentration is exhibited in Fig. 3. With the increase of PM_2.5_ concentration, the heart rate has an overall upward trend despite some fluctuations. For male youth group, the heart rate is significantly lower than other groups and increases linearly. The heart rate in female middle-aged group is the highest. Its growth trend is similar to that of female youth group and male middle-aged group with an "S" curve. Two inflection points on the curve represents PM_2.5_ concentrations of 100ug/m^2^ and 180 ug/m^2^, respectively. The regression curves for male middle-aged group and female youth group have an intersection point. The heart rate of male middle-aged group is higher than that of female youth group when PM_2.5_ concentration is below 220 ug/m^2^. While PM_2.5_ concentration is above 220 ug/m^2^, the reverse applies. On the whole, youths are better at regulating the pollutant effects on body function. The physical performance of males are more stable than females as PM_2.5_ concentrations increase.

Table 3 shows the heart rate statistics of volunteers in each group. The average heart rate in female middle-aged group is about 5% higher than that of female youth group and about 9% higher than male youth group. The average heart rate is approximately equally represented in female youth group and male middle-aged group with 1% gap. Middle-aged volunteers maintain a higher heart rate and smaller room to adjust in the polluted environment. In contrast, youths appear better adaptability which is represented by higher standard deviations, i.e., larger adjustment room.

**Table 3:** Statistics of heart rate indexes.

Group	Mean	Standard deviation	Maximum	Minimum
Male youth group	80.69	10.86	112	58
Female youth group	84.14	10.29	125	59
Male middle-aged group	85.29	9.78	124	60
Female middle-aged group	88.25	9.18	126	59

### Determination of health risk thresholds

In the experiment, the heart rate varied from person to person due to differences in basic physical conditions and their status on that day. To avoid this kind of influence, HR% is adopted to characterize heart rate changes for different volunteers and different experiment dates. 10 min before the experiment, the heart rate was measured in a room with air purification and its mean value was taken as the heart rate in healthy state. HR% is expressed by the formula (4):

4 $$r_{i} = \frac{{t_{i} - t}}{t}$$

where: *r*_*i*_ is the average change of heart rate during time interval *i*; *t* is the average heart rate measured 10 min before the experiment; *t*_*i*_ is the average heart rate during time interval *i*.

**Figure 4:**
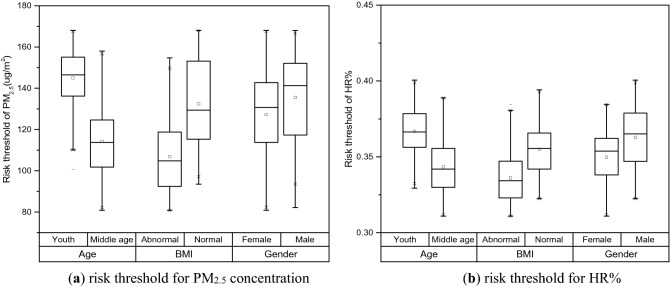
Distribution of health risk thresholds among volunteers.

In this research, ROC curve is applied in order to calculate the threshold of health risk. The input state variable is HR% and the test variable is subjective judgment of safety and risk for volunteers. Safe state takes 0 as the value and 1 represents risk state. Possible thresholds should be selected at first, then the sensitivity and specificity of each threshold are calculated. Based on the value range of HR%, 0.005 is determined as the discriminant interval of threshold. For 337 out of 368 volunteers, the area under ROC curve is larger than 0.9, indicating high accuracy of the experiment. With the increase of PM_2.5_ concentration, HR% gradually rises. The existing of fluctuations leads to the situation that HR% at the optimal critical point may correspond to multiple PM_2.5_ concentration values. The solution is to take the average of these values as the threshold for safe and risk state. The thresholds for volunteers under different physical conditions are shown in Fig. 4.

HR% at the optimal threshold of health risk ranges from 31.1% to 40.1%, corresponding to a PM concentration range of 81 to 168 ug/m^2^. For male volunteers, the average concentration at which risk occurs is 136 ug/m^2^. It is higher than that for females (127 ug/m^2^), indicating that males show greater resistance to air pollution. The risky concentration for youth is 145 ug/m^2^, significantly higher than 114 ug/m^2^ for middle aged people. As people age, the hypofunction results in lower receptivity to the external environment. If a volunteer’s BMI stays in the normal (healthy) section, his (her) risky PM concentration is 132 ug/m^2^, which is higher than 107 ug/m^2^ in abnormal (non-healthy) section. It proves that physical condition will affect the reaction to the external environment. Good physical quality would increase the adaptability and improve pollution resistance.

### Health risk characteristics

The occurrence of health risks is correlated with HR%, pollution exposure concentration, gender, age and BMI. In order to avoid the error caused by high correlation of these explanatory variables, a correlation test should be undertaken before modeling. Pearson correlation coefficient was utilized in this research. Pearson correlation coefficient takes values between 0 and 1. It can be divided into five intervals with an interval step of 0.2. The correlation coefficient (from large to small) indicates the correlations are very strong, strong, moderate, weak and very weak. Correlations between different explanatory variables are listed in Table 4.

**Table 4:** Correlation of explanatory variables.

	HR%	PM_2.5_	Gender	Age	BMI
HR%	1	0.386	0.337	0.261	0.355
PM_2.5_	0.386	1	0.053	0.021	0.102
Gender	0.337	0.053	1	0.034	0.064
Age	0.261	0.021	0.034	1	0.217
BMI	0.355	0.102	0.064	0.217	1

**The correlation was significant at the 0.01 level (two-tailed).

As illustrated in Table 4, there is no significant correlation between explanatory variables. Thus, five variables (PM_2.5_ concentration, gender, age, BMI and HR%) can be selected as explanatory variables. The occurrence of health risks is correlated with HR%, pollution exposure concentration, gender, age and BMI. According to the threshold of health risk, the status of volunteers is classified into two categories: safe state and risk state. Then binary logit model is established.

5 $$\ln \left( {\frac{p}{1 - p}} \right) = \beta_{0} + \beta_{1} \gamma_{1} + \beta_{2} \gamma_{2} + \beta_{3} \gamma_{3} + \beta_{4} \gamma_{4} + \beta_{5} \gamma_{5}$$

where: *p* is the health risk level; $\gamma_{1}$ is the concentration of PM_2.5_; $\gamma_{2}$, $\gamma_{3}$, and $\gamma_{4}$ are 0–1 dummy variables ($\gamma_{2}$: 1-male, 0-other; $\gamma_{3}$: 1-youth, 0-other; $\gamma_{4}$:1-BMI in healthy section, 0-other); $\gamma_{5}$ is HR% with an interval of 60 s; $\beta_{0}$ is the undetermined coefficient; $\beta_{1}$ ~ $\beta_{5}$ are the coefficients of $\gamma_{1}$ ~ $\gamma_{5}$ variables, respectively.

The comprehensive analysis results for each parameter are shown in Table 5. The significance levels of all variables are less than 0.05, which proved the validity of the model. Among all coefficients of variables, $\beta_{1}$ and $\beta_{5}$ are positive and the remaining are negative. It indicates that the higher the PM_2.5_ concentration and HR%, the higher the probability of generating health risks. Males, youth and those with BMI in normal section are more adaptable to polluted weather. The analysis results from model are consistent with statistical results from experiment.

**Table 5:** The value of model parameters (HR%, PM_2.5_ concentration, gender, age and BMI).

Variables	$\beta_{j}$	Standard Error	Wald test	Degree Freedom	P value	Exp ($\beta_{j}$)
$\gamma_{1}$	0.056	0.578	68.578	1	0.000	1.058
$\gamma_{2}$	− 0.264	0.275	6.235	1	0.023	0.768
$\gamma_{3}$	− 0.393	0.069	29.189	1	0.008	0.675
$\gamma_{4}$	− 0.327	0.322	12.624	1	0.011	0.721
$\gamma_{5}$	0.108	0.396	17.591	1	0.016	1.114
$\beta_{0}$	− 6.323	0.821	39.467	1	0.005	0.002

The statistical value is the mean value of the parameters.

The coefficient of PM_2.5_ concentration is 0.056 and Exp ($\beta_{{1}}$) is 1.058, indicating that for each unit increase in concentration, the probability of risk will be 1.058 times higher. The coefficient for males is − 0.264 and Exp ($\beta_{{2}}$) is 0.768, proving that the probability of risk for males is 76.8% of that for females. The coefficient for youth is − 0.393 and Exp ($\beta_{{3}}$) is 0.675, indicating that the probability of risk for youth is 67.5% of that for middle aged people. The coefficient for people whose BMI falls in normal interval is − 0.327 and Exp ($\beta_{4}$) is 0.721. It shows that the probability of risk for healthy volunteers is 72.1% of that for non-healthy ones. The coefficient of HR% is 0.108 and Exp ($\beta_{5}$) is 1.114. It means that for each unit increase in HR%, the probability of risk will be 1.114 times greater.

Wald test is a method to estimate the significance of a given explanatory variable in a statistical model^35^. Its value takes the ratio between the regression coefficient and the standard error. A higher Wald value means a more essential explanatory variable. In this research, PM_2.5_ concentration and age have the largest Wald test values and both significance levels are less than 0.05. The explanation is that these two variables have a significant effect on the health risk degree. The Wald test value of PM_2.5_ concentration is significantly higher than other variables, demonstrating that it has the greatest impact.

According to the analysis results, the health risk model is calibrated as:

6 $$\ln \left( {\frac{p}{1 - p}} \right) = - 6.323 + 0.056\gamma_{1} - 0.264\gamma_{2} - 0.393\gamma_{3} - {0}{\text{.327}}\gamma_{4} + 0.108\gamma_{{5}}$$

Based on formula (6), the health risk degree *p* can be calculated:

7 $$p = \frac{{\exp ( - 6.323 + 0.056\gamma_{1} - 0.264\gamma_{2} - 0.393\gamma_{3} - {0}{\text{.327}}\gamma_{4} + 0.108\gamma_{{5}} )}}{{1 + \exp ( - 6.323 + 0.056\gamma_{1} - 0.264\gamma_{2} - 0.393\gamma_{3} - {0}{\text{.327}}\gamma_{4} + 0.108\gamma_{4} )}}$$

**Figure 5:**
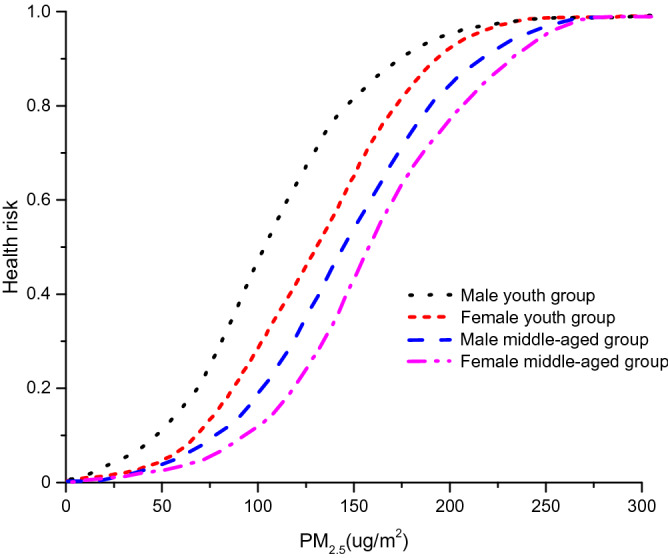
The probability of health risks in each group of volunteers as PM_2.5_ concentration increases.

The health risk degree is calculated respectively for each group of volunteers. Final results and health risk curves are shown in Fig. 5. As PM_2.5_ concentrations increase, the probability of health risk gradually comes up. Striking differences are exhibited between males and females, youth and middle-aged people. It is apparent that males and youth have better resistance to pollution. At the same PM_2.5_ concentration, the probability of health risk from high to low in order is: female middle-aged group > male middle-aged group > female youth group > male youth group.

## Discussion

Epidemiological and toxicologic studies have shown that PM_2.5_ can induce cardiovascular and respiratory diseases. Moreover, it has many other adverse effects on human health, such as Type 2 Diabetes, birth defects and premature deaths. The underlying mechanisms of the relationship between PM_2.5_ and poor health include the oxidation reactions in induced cells, mutagenicity and inflammation^36^. Research results demonstrate that exposed environment with high PM_2.5_ concentration will lead to increasing probability of commuters’ health risks, which is consistent with former conclusions^37–39^. When the sudden change in heart rate reaches 35% or more, it means that there is a large external environmental disturbance and the body's regulatory function starts to feedback^21^. When the concentration of pollutants in the air is higher than 150 μg/m^3^, the body's blood oxygen saturation is below the normal range and health risks occur^40^. The above findings lie within the health risk threshold of this paper.

The youth and the commuters with BMI index in normal section have better resistance to pollution. The probability of risk for youth is 67.5% of that for middle-aged people, and the probability of risk for people with BMI in healthy range is 72.1% of that for people with BMI in non-healthy range. Compared with middle-aged people, the youth have greater capacity to adapt to the external environment. This phenomenon is mainly related to the sensitivity of body functions to external reactions. Existing researches have shown that pollutants can activate the youth’s cardiorespiratory functions^41,42^, and youths have 1.3 times more space for cardiorespiratory conditioning than middle-aged^43^. Both obesity and thinness have an impact on body resistance to the external environment. In epidemiological studies, BMI index is highly correlated with anti-pollution ability. For obese people, long-term exposure to pollutants will increase their insulin production and then rise blood sugar levels^44^. People with lower BMI index tend to have poor immunity. So, their risks of coronary heart disease and hypertension caused by air pollution is 1.2 times higher than that for people with normal BMI index. As a result, the death rate is about 1.1 times higher than normal value^45,46^.

An unexpected but interesting finding is the sex-specific difference of health risks under air pollution environment, which is reflected in better resistance to pollution for males. In the same travel environment, the probability of risk for male is 76.8% of that for female. This conclusion indicates that some unknown factors associated with sex or gender affect the body's response to pollutants. People are becoming more and more interested in the role of sex and gender played in the epidemiology relevant to air pollution. Several recent studies have reported that pollutants have a greater impact on female’s respiratory health than male^47,48^. A comprehensive analysis of the relationship between pollutants and death rate found that in a highly polluted environment, the risk for females increased by 1.12% and that for males increased by 0.73%^49^. However, in epidemiological research, it is complicated to draw a distinction between sex (e.g., physiology, toxicokinetics) and gender (e.g., the nature of work, disease-related behavior, activities in males and females). It is still not clear whether the biology or behavioral/social differences drive the discrepancy found in this study.

The change of sensitivity to PM_2.5_ may be related to physiological differences caused by hormonal, structural and morphological differences between males and females. Specifically, it is known that males and females differ in lung size, airway diameter, air absorption and cardiovascular response. These differences may be a significant mechanism that directly affects commuter’s inhalation dose of PM_2.5_, which eventually leads to different health risks^50,51^. Toxicokinetic differences in the absorption and metabolism of PM_2.5_ components can affect the acceptable pollutant-related dose for males and females. Males and females have different air absorption capabilities, and the permeability of the gas-blood barrier differs by sex^52^. The inflammatory response is another important mechanism that is considered as an intermediate step in the link between air pollution and health^53^. Sexual differences in antioxidant status may have different effects on inflammatory and oxidative stress processes in males and females. However, it is not clear whether these responses biologically favor males or females.

Researches on air pollution emphasize sex-based differences when analyzing various health effects. But it is not clear whether these effects are from sex-based differences, gender-based differences, or both. The above review provides several explanations for different commuting health risks based on PM_2.5_ exposure, but the direction of these effects is not always clear. Although a large number of studies have shown that females are more susceptible to outdoor air pollution, some toxicological studies suggest that males may be more susceptible due to higher doses of inhaled pollutants. Future researches should continue to investigate the sex-based differences in air pollution exposure. Collecting data on variables (activity pattern, occupations, income, socioeconomic status and etc.) that can be used to replace gender differences should be also focused. This study was able to evaluate sex-based differences but lacked information on gender or gender substitution variables.

## Conclusion

Based on ECG data and PM concentration data, this study applies ROC curve to calculate the threshold of health risk during walking trips. Then a binary logit model that integrates pollutant concentration, gender, age and BMI is established. The probability of risk during trips can be effectively obtained by the proposed method. When PM_2.5_ concentration is in the interval of 0–300ug/m^2^, HR% gradually increases along with the rising of PM concentration. Males, youth and those with healthy BMI have better pollution resistance, as evidenced by having a higher risk threshold. Each unit increase in PM_2.5_ concentration, the probability of risk will be 1.058 times higher; each unit increase in HR%, the probability of risk will be 1.114 times greater; the probability of risk for males is 76.8% of that for females; the probability of risk for youth is 67.5% of that for middle aged people; the probability of risk for normal BMI volunteers is 72.1% of that for abnormal ones. Among all indicators, PM_2.5_ concentration and age impact the health risk degree more significantly. The greatest one is PM_2.5_ concentration. The research is limited to the health risk of walking. While residents' travelling can be accomplished through a variety of other transportation modes, which requires further study.
